# An Account of the Practice of One of the Physicians of the Westminster General Dispensary, and of the Western Dispensary, from the 20th of February to the 20th of March, 1807

**Published:** 1807-04

**Authors:** Samuel Fothergill

**Affiliations:** Southampton Street, Strand


### *An Account of the Practice of one of the Physicians of the Westminster General Dispensary, and of the Western Dispensary, from the 20th of February to the 20th of March,* 1807.

| | |
|---|---|
| **ACUTE DISEASES.** | |
| Typhus - - - | 3 |
| Acute Rheumatism - | 4 |
| Peripneumony - - | 2 |
| Inflammatory Sore Throat | 2 |
| Catarrh - - - | 6 |
| Acute Diseases of Infants | 12 |
| **CHRONIC DISEASES.** | |
| Cough and Dyspnœa - | 40 |
| Pleurodyne - - | 3 |
| Pneumonia Notha - | 4 |
| Pulmonary Consumption | 4 |
| Hæmoptoe - - - | 3 |
| Chronic Rheumatism | 10 |
| Lumbago - - - | 5 |
| Asthma - - - | 4 |
| Asthenia - - - | 12 |
| Paralysis - - - | 3 |
| Cephalalgia - - | 2 |
| Gastrodynia - - | 5 |
| Constipation - - | 3 |
| Diarrhœa - - - | 4 |
| Dyspepsia - - | 6 |
| Hæmatemesis - - | 2 |
| Jaundice - - - | 3 |
| Hypochondriasis - | 2 |
| Worms - - - | 3 |
| Cutaneous Diseases - | 7 |
| Hysteria - - - | 2 |
| Amenorrhœa - - | 5 |
| Menorrhagia - - | 6 |
| Leucorrhœa - - | 4 |
| Abortion - - - | 2 |

The case of Tic Douloureux recorded in last month's Report, has terminated favourably. This disease rarely comes under our notice, and as it is seldom cured without an operation, I have considerable satisfaction in giving a brief statement of the case alluded to.

Mrs. P. aged 33, about ten years ago, whilst sitting at dinner, was suddenly seized with an acute pain, darting through the right cheek; it presently ceased, but returned again every day about the same time. From that period till now, (January 24) she has seldom passed many days together without an attack, which sometimes continues with little intermission for several hours, becomes more violent towards evening, and affects the whole cheek, ala nasi, nasi, shoots into the gums, and is sometimes felt about the supercilium and temple of the same side. She has had two teeth extracted, and has taken several medicines without having received any essential benefit. Opiates given in large doses have procured little ease; and from the quantity taken, have produced too unpleasant effects to be increased.

I directed her to take five grains of extract of hemlock, three times a-day. This medicine, either from a difference in the preparation or the quality, or from some peculiarity of constitution, seems to produce very different effects; sometimes having an evident influence upon the system, and sometimes none whatever; in almost all cases it may be gradually increased, till the patient can bear a very large quantity; if it is then omitted, and after some cessation, the same dose is given, it will sometimes prove fatal; an instance of which is related by Mr. John Hunter.

Mrs. P. continued the cicuta ten days, the dose being increased to ten grains four times a-day; no sensible effect was produced, and the pain continuing violent, Fowler's solution of arsenic, with equal parts of laudanum, was given; she began with ten drops three times a-day, and in two weeks, gradually increased the dose to fifteen drops every six hours. About this time the bowels became affected with diarrhœa and griping pains, without the complaint being in the least relieved. The arsenic solution was consequently omitted, and decoction of bark, with the caustic volatile alkali was now given every six hours. In two days she began to experience some ease; and in one week more was entirely free from pain, and expressed herself with great joy, to be as well as she was before her first attack. She has been married two years and a half, and her husband informs me, she has scarcely passed a-day without suffering severely from her complaint. Her face was never swelled; but sometimes during the continuance of the pain was red, and the eye slightly inflamed and watery.—Her appetite was pretty good; but her strength was sinking, and she laboured under considerable anxiety of mind, from the long continuance of her illness. She has taken no medicine during the last three weeks, and has continued free from any accession of pain.

In this case, it may be doubted, whether the bark or the caustic alkali, produced the favourable change in the symptoms. Cinchona has been given in Tic Douloureux without any good effect; and I am rather disposed to ascribe cribe it to the caustic alkali; which I gave from having heard that it has been very successful in restoring people who have been bitten by the cobra de capello, the poison of which, seems to act immediately on the nerves.

As the above, however, is the only instance of Tic Douloureux, in which I have known this medicine to be administered, I shall refrain from reasoning upon a single case; but should be highly gratified to hear of similar success attending its exhibition by any other practitioner.

SAMUEL FOTHERGILL.

*Southampton Street, Strand.*

